# Retrospective study of disparities in regional anesthesia and discharge opioid prescriptions at a veterans affairs medical center

**DOI:** 10.12688/f1000research.139684.1

**Published:** 2023-11-08

**Authors:** Mercy A. Udoji, Oluwatoyin Thompson, Xiangqin Cui, Kathryn E. Glas, Anna Woodbury

**Affiliations:** 1 Department of Anesthesiology and Pain Medicine, Emory University, Atlanta, Georgia, USA; 2 Emory University, Atlanta, Georgia, USA; 3 Department of Biostatistics and Informatics, Emory University, Atlanta, Georgia, USA; 4 Department of Anesthesiology, The University of Arizona, Tucson, Arizona, USA

**Keywords:** Veterans health, chronic pain, total knee arthroplasty (TKA), disparities, opioids, regional anesthesia

## Abstract

**Background:** Abundant literature acknowledges healthcare disparities exist in medicine, especially in pain management, but disparities related to peri-operative pain management in veterans undergoing total knee arthroplasties (TKA) has not been previously described. TKAs are becoming increasingly common, and evidence suggests that perioperative regional anesthesia improves post TKA outcomes. This study aimed to determine if healthcare disparities exist pertaining to the use of regional anesthesia and the prescribing of discharge opioids for TKAs in the Veterans Affairs Health Care System (VAHCS). We hypothesized that race-based disparities would be present in the use of regional anesthesia and discharge opioid prescribing at our institution. Our secondary hypothesis was that older patients would be more likely to receive regional anesthesia and lower quantities of opioids at discharge.

**Methods:** This was a retrospective analysis of Atlanta VAHCS patients who underwent elective unilateral primary or revision TKA surgery between 2014 and 2020. A total of 653 patients were included. Multivariate logistic regression was used to model the impact of patient demographics on nerve block use and multivariate linear regression was used to model the impact of patient demographics on total oral morphine equivalents prescribed.

**Results:** Our results showed that Black patients were as likely to receive regional anesthesia for their TKAs (p=0.85) but did receive less opioid pain medications at discharge (p<0.001) than White patients. We also found that older patients ( > 50 years old) had significantly lower odds ratio of receiving regional anesthesia and received less opioid pain medications post TKA discharge.

**Conclusions:** Our study showed age-based disparities in regional anesthesia utilization and discharge opioid prescriptions. It also showed race-based disparities in discharge opioid prescriptions. Our results demonstrate the need to better understand why these differences exist within this open access system and suggests solutions based on the socioecological model to diminish them.

## Glossary of terms

• Atlanta Veterans Affairs Health Care System (AVAHCS)
• Veterans Affairs Health Care System (VAHCS)
• Veterans Health Administration (VHA)
• United States (US)
• Oral Morphine Equivalent (OME)
• Total Knee Arthroplasty (TKA)
• Regional Anesthesia (RA)
• Neuraxial Anesthesia (NA)
• Peripheral nerve block (PNB)
• Current Procedural Terminology (CPT)
• Strengthening the Reporting of Observational Studies in Epidemiology (STROBE)

## Introduction

Care delivered by the United States’ (US) health care system differs based on race, age, and sex ^1^ and customarily results in diminished clinical outcomes for those patients affected. The Veterans Health Administration (VHA), the largest health care system in the United States, has traditionally been thought to be somewhat insulated from these differences in care because of its open access structure and single insurer. However, the data shows that significant health care disparities still exist within that system. ^2^^–^^4^

Race-based disparities in opioid prescribing for acute and chronic pain are well described. ^5^^–^^8^ These disparities have been attributed to a variety of causes such as pain estimation biases, access issues amongst minorities, sex and racial differences in pain expression, and bias from health care workers. ^6^^,^^9^^,^^10^

Total knee arthroplasties (TKAs) are one of the most common operations performed in the US and is expected to grow to more than 900,000 per year by 2030. ^11^ There is a literature supporting the fact that regional anesthesia (RA) in the form of peripheral nerve blocks (PNB) and neuraxial anesthesia (NA) improves post TKA outcomes such as length of stay, decreased post operative opioid consumption, and infection rates *and* that it is underutilized in Black patients. ^12^^–^^14^ Studies also suggest that high levels of pain post operatively may be associated with increased risk of developing chronic post-surgical pain. ^15^

Despite all we know about disparate pain care, there is limited research exploring disparities related to postoperative discharge prescriptions, ^7^^,^^16^ especially within the VHA as it delivers care to veterans. Our study sought to fill that gap.

Our aim was to determine whether disparities related to the use of regional anesthesia and the prescription of discharge opioids for TKA patients exist within the Atlanta VAHCS (AVAHCS). Prior to data collection, we hypothesized that Black patients would receive regional anesthesia for TKA less often than White patients and that Black patients would receive lower amounts of opioids for post discharge consumption. Our secondary hypothesis was that older patients would be more likely to receive RA and lower quantities of opioids at discharge.

## Methods

This research study was not preregistered at an independent registry.

### Study design and ethical statement

This study was approved by the institutional review board (IRB) of Emory University and Atlanta Veterans Affairs Medical Center (approval #1857, date of approval December 7, 2020). The requirement for written informed consent was waived by the IRB due to the non-interventional retrospective design the study. This study adhered to the applicable Strengthening the Reporting of Observational Studies in Epidemiology (STROBE) guidelines. ^17^

### Patient selection and data extraction

We conducted a single center retrospective review of all adult patients who underwent total knee arthroplasties for end-stage knee osteoarthritis at the Atlanta Veterans Affairs Medical Center from January 1, 2014 through December 31, 2020. This timeframe was chosen to try to capture as many patients as we could while still avoiding bias due to practice changes in anesthesia. We also chose the end date of December 2020 due to the COVID pandemic and resultant significant decrease in # of surgeries performed at our institution in 2020. To mitigate bias, everyone who had a TKA (primary or revision) during that time were included in the initial data pull. We assessed 846 patients who met that definition during the period of interest for eligibility. Our exclusion criteria were as follows: patients <18 years of age, bilateral TKA, TKA with ligament repair, partial arthroplasty, patients discharged to nursing homes or rehabilitation facilities or other inpatient setting (and therefore were not prescribed opioids at discharge). For those who underwent more than one TKA during the study period, only data from their first operation was included to avoid confounding.

The following variables were extracted from the records: patient demographics (sex (self reported) race, age), surgery type (primary versus revision TKA), receipt of a peripheral nerve block (PNB) or neuraxial anesthesia (NA), name and quantity of discharge opioids, diagnosis and procedure codes, and admission status. The following Current Procedural Terminology ^®^ (CPT) codes were used in our query to identify TKA patients: 27440, 27446, 27445, 27447, 27486, 27487. The following codes were used to identify patients who received any kind of regional anesthesia for their operation: 62326, 62322, 64447, and 64448. Popliteal or sciatic nerve blocks are not used at our institution for post-TKA pain management therefore the codes for sciatic/popliteal nerve blocks were not included in our query.

For patients in which surgery type was unclear after the initial query (for example multiple CPT codes were entered in the surgical package on the date of surgery suggesting that TKA and another procedure such as a meniscus repair was performed concomitantly), M.A.U reviewed patient charts to determine if inclusion criteria were met. Discharge oral morphine equivalents (OMEs) were calculated based on opioid-type medications that were dispensed three days before to three days after day of surgery because the average length of stay post-TKA was less than two days at our institution. When more than one opioid prescription was identified in the period of interest, M.A.U reviewed the charts in question to determine which prescriptions should be included (for example, some patients received refills of opiates for chronic pain, separate from their discharge prescriptions for acute postoperative pain; others received partial fills and received the rest of their prescriptions a day later). Please see Table 1 for the opioid conversion factors used in this study.

**Table 1.** Opioid conversion factors utilized for OME calculation. ^40^

Opioid	Conversion factor (mg/OME)
Hydrocodone	1
Oxycodone	1.5
Codeine	0.15
Hydromorphone	4
Morphine	1
Meperidine	0.1
Tramadol	0.1

OME=oral morphine equivalents.

### Statistical analysis

The national VA corporate data warehouse was queried using the parameters above. Data collected was cleaned before statistical analysis. That process involved removing patients who had more than one TKA operation during the time of interest as stated above. It also involved reviewing charts when more than one procedure code was used and excluding those patients. Lastly, we removed data from patients who either did not receive opioids at discharge or those who were discharged to a facility (rehabilitation or nursing home) as they were not prescribed opioids by VA based physicians in the OME related analysis. After, summary statistics were generated for all the variables. Multivariate logistic regression was used to model nerve block use (yes/no) outcome. Multivariate linear regression was used to model total OMEs. Odds ratios and mean difference estimates were calculated, a 95% confidence interval was produced. Significance level was set at a p value of 0.05. All statistical analyses were conducted using R version 4.1.2.

## Results

Our initial data query revealed 846 veterans were eligible for the study. After the exclusion criteria (described in detail in the “methods” section above) were applied, six hundred and fifty-three veterans were included in the analyses. Table 2 shows the demographics and baseline characteristics of our cohort. Sex-based analyses were not performed because of the low proportion of female veterans in our cohort.

**Table 2.** Baseline characteristics of the study cohort.

	Overall N=653
Age at surgery (years)	
• 0-49	**•** 47 (7.2%)
• 50-64	**•** 321 (49.2%)
• ≥65	**•** 285 (43.6%)
Race	
• White	**•** 278 (42.6%)
• Black	**•** 335 (51.3%)
• Other	**•** 5 (0.8%)
• Unknown	**•** 35 (5.4%)
Sex	
• Male	**•** 579 (88.7%)
• Female	**•** 74 (11.3%)
TKA type	
• Primary	**•** 529 (81%)
• Revision	**•** 124 (19%)
Total OME	
• Mean (SD)	**•** 672 (393)
• Median [Min, Max]	**•** 675 [0, 3240]

TKA=total knee arthroplasty, OME=oral morphine equivalents, SD=standard deviation.

When the effect of patient demographics on regional anesthesia utilization was examined via multivariate logistic regression analysis, no statistically significant differences based on race (p=0.85), sex (p=0.36), or surgery type (p=0.86) were found. Surprisingly, increased age (50 or greater) was associated with lower odds ratio of receiving any type of nerve block for a TKA (p=0.01 for patients aged 50-64; p<0.05 for patients ≥ 65) when compared to patients 49 years of age or younger ( Table 3).

**Table 3.** Multivariate logistic regression examining effect of demographics on use of regional anesthesia.

Predictor	Odds Ratio (95% CI)	P Value
**Age at surgery**		
Under 50	Reference Group	
50-64	0.37 (0.16, 0.77)	*0.01*
≥65	0.44 (0.19, 0.95)	*<0.05*
**Race**		
White	Reference Group	
Black	1.03 (0.73, 1.47)	0.85
Other	0.26 (0.03, 1.74)	0.17
Unknown	1.40 (0.66, 3.18)	0.39
**Sex**		
Female	Reference Group	
Male	0.77 (0.44, 1.33)	0.36
**TKA Type**		
Primary	Reference Group	
Revision	0.96 (0.64, 1.47)	0.86

Note: P values that are significant (<0.05) have been italicized.

TKA=total knee arthroplasty, CI=confidence interval.

Our dataset included 593 patients who received opioid type pain medications at the time of their discharge. When discharge opioid prescriptions were assessed via multivariate linear regression analysis, it revealed that Black patients received less pain medications (109.64 (p<0.001) lower OMEs) for post-surgical pain when compared to White patients ( Table 4). The effect of age on discharge opioid prescription was examined and the data demonstrated that patients aged 50 or greater received less opioids at discharge as compared to younger patients. Those aged 50-64 received 149.25 lower OMEs (p=0.01) while those ≥65 received 252.95 (p<0.001) lower OMEs.

**Table 4.** Multivariate linear regression examining the effects of patient demographics on discharge opioid prescription.

Predictor	Mean difference estimate (95% CI)	P Value
**Age at surgery**		
Under 50	Reference Group	
50-64	-149.25 (-268.99, -29.51)	*0.01*
≥65	-252.95 (-378.27, -127.64)	*<0.001*
**Race**		
White	Reference Group	
Black	-109.64 (-174.40, -44.87)	*<0.001*
Other	62.52 (-282.60, 407.65)	0.72
Unknown	-93.39 (-228.66, 41.87)	0.18
**Sex**		
Female	Reference Group	
Male	14.40 (-82.73, 111.54)	0.77
**TKA type**		
Primary	Reference Group	
Revision	-123.38 (-199.73, -47.02)	*<0.01*

Note: P values that are significant (<0.05) have been italicized.

TKA=total knee arthroplasty, CI=confidence interval.

Finally, discharge opioid prescriptions for primary versus revision TKA were compared and revealed that patients undergoing revision TKAs received lower amounts of opioids (-123.38 (p<0.01) lower OMEs) at discharge than primary TKA patients. There were no statistically significant differences in discharge opioid prescriptions for patients whose race were categorized as Other/Unknown or when sex was considered.

## Discussion

Our single center retrospective review is the first to assess for disparities in RA utilization and post-surgical opioid prescriptions within the VAHCS. It revealed race-based disparities in discharge opioid prescriptions and age-based disparities in RA utilization for TKAs.

*Regional anesthesia*: The VAHCS is the largest health care system in the US serving a population that data shows experience chronic pain at a higher rate than the general population. ^18^ Up to 34% of patients develop chronic post-surgical pain (CPSP) after TKA and perioperative pain has been put forth as a modifiable risk factor. ^15^^,^^19^ Chief among the many benefits of using RA for TKA is reduction of post-surgical pain; therefore, disparate use of RA places those affected patients at higher risk of CPSP and the resultant decrement of quality of life and increased utilization of healthcare resources.

Factors ranging from hospital type and setting, payor considerations and availability of RA services have been used to explain RA disparities ^12^; however, those factors do not play a role within the VAHCS. Contrary to other studies, we found that advanced age was associated with decreased use of RA. Overrepresentation of Black patients (51% versus 13% in the general population) ^20^ may have skewed our results because Black patients agree to RA less often than other racial groups. ^21^^,^^22^ Reasons for avoidance of RA in older patients might be the presence of comorbid conditions necessitating use of anticoagulants and fear of side effects.

*Post-operative discharge opioids*: Our finding of race-based disparities in discharge opioids was disappointing but consistent with the literature showing that black patients are prescribed less opioids for pain. ^6^^,^^7^^,^^23^ Bias is thought to contribute significantly to disparate pain care especially as it pertains to race and sex. ^9^^,^^24^ Age-based reduction in discharge OMEs was also not an unexpected finding but revision TKA patients receiving lower OMEs at discharge was. Reasons for this result may be that revision patients were older or reported lower perioperative pain scores.

Our study adds to the literature that demonstrates the pervasive nature of racial disparities in healthcare but is unique in highlighting disparities in postoperative opioid prescribing and use of regional anesthesia that may exist within the VAHCS.

**Figure 1.**
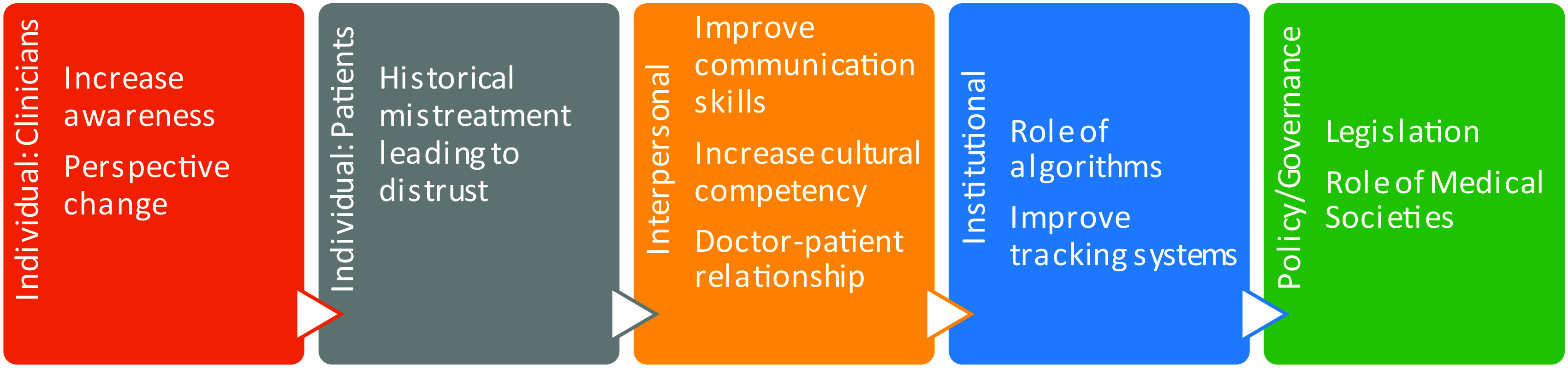
Modified Socioecological Approach to Disparities in Pain Care: This figure demonstrates suggested approaches to reduce pain care disparities from the individual (patient and clinician) to institutional to governmental perspectives. This figure is an original figure produced by the authors for this article.

Disparate care is a multi-faceted problem and should be approached in a similarly multi-faceted manner to drive change. A modified socioecological model provides a framework within which this issue can be addressed ( Figure 1).

*Individual➔clinician* : Accepting that implicit bias perpetuates disparate care is important. Despite the evidence demonstrating clinicians hold stereotypical thoughts about Black patients, ^25^^,^^26^ a common reaction to bias training is denial, defensiveness, and a focus on *intent* rather than *outcome.* To better understand and reduce the role of implicit bias in the care that we provide, clinicians are urged to educate themselves via resources like the Implicit Association Test. ^27^ Educational institutions can also include bias training as part of their medical education curriculum. Other “empathy-inducing interventions” such as perspective taking has been shown to mitigate implicit bias. ^28^

*Individual➔patient:* Black Americans’ mistrust of the medical system is deeply seated in history dating from the antebellum period and Dr. Marion Sim’s horrific experimentation on Black women to the more recent Mississippi Appendectomy. ^29^ This history and readily available information (via social media and other sources) about the magnitude and impact of race-based disparities informs the way Black patients view healthcare systems and increases psychological distress. ^30^^,^^31^ Combating misinformation about RA ^22^ and increasing diversity in the healthcare workforce may partially ameliorate these differences.

*Interpersonal* : Chronic pain research suggests that disparities are often perpetuated by the patient-physician relationship. ^32^ Black patients tend to rate clinical visits lower for interpersonal care when compared to White patients. ^31^ Increasing our cultural competency and awareness, communication skills, and providing patient-centered care may lead to more effective communication, better interactions with patients, reduced bias, and improved patient confidence in care that is provided. ^33^^,^^34^

*Institutional/health care systems* : Health care systems can reduce disparities by investing in community outreach programs for patient education and to build trust. Implementing algorithms clinically can also mitigate disparate care since data from enhanced recovery programs ^35^ demonstrate elimination of disparities post-implementation. Healthcare systems can support these measures by funding financial and time-based resources for staff education and track equitable delivery as part of existing quality improvement processes.

*Policy/governance:* Attempts are being made to leverage the power of legislation to address the issue of health care disparities. A recent executive order entitled “Advancing Racial Equity and Support for Underserved Communities Through the Federal Government” ^36^ and the CDC’s “Healthy People 2020” ^37^ program are two examples. The Agency for Healthcare Research and Quality also produces an annual report entitled the “National Healthcare Quality and disparities Report” outlining the scope and effect of disparate health care. ^38^ Similarly, the American Medical Association has written whitepapers on health care disparities and produced a Disparities Toolkit to assist clinicians in tackling this issue. ^39^ It is unclear the effect the preceding has had on care received at the bedside.

*Study limitations:* Our study design is retrospective with potential for inherent bias, limited number of patients, limited surgery type, and only included one urban VA Medical Center. There was no attempt made to account for practice change over time especially as it relates to opioid prescribing. Our study population was also sex imbalanced which may not have allowed us to recognize differences in the care provided to female veterans. Lastly, pain scores and pre-operative OME data which may have played a role in the provision of RA and the amount of discharge opioids prescribed was not collected by our team as part of this study.

*Future directions:* In the future, we hope to acquire funding to query the system-wide data warehouse to corroborate our results and evaluate other factors such as perioperative pain scores and preoperative medication use that may have skewed them. At our hospital, a standard algorithm for multimodal analgesic regimen for TKA patients was implemented soon after the results of this study was available, and work is being done to standardize post operative opioid prescribing.

## Data Availability

Access to underlying data for this study is not able to be provided as our IRB does not allow the deposition of this dataset consisting of veterans to be deposited or dispersed in any way outside the VHA firewall whether it is de-identified or not. The data underlying the results can be accessed via a Data Access request made to the VHA informatics and computing infrastructure after approval of the IRB of the Emory University School of medicine and the Atlanta Veterans Affairs Medical Center ( https://www.irb.emory.edu).
