# Supplementary material for: Retrospective study of disparities in regional anesthesia and discharge opioid prescriptions at a veterans affairs medical center
Source: F1000Res. 2023 Nov 8;12:1451. [Version 1] doi: 10.12688/f1000research.139684.1 (PMC11803443; doi:10.12688/f1000research.139684.1)
Supplement: Peer review history

# Reviewer report: Reviewer response for version 1

**DOI:** 10.5256/f1000research.152981.r306080

**Published:** 2024-12-30

**Authors:** Channing Twyner

**Affiliations:** 1 University of Mississippi Medical Center, Jackson, MS, USA

This manuscript asks readers to consider a socioecological framework to address health disparities using variation in access to opioids and regional anesthesia as an example.  Specifically, African American race was associated with decrease access to post operative discharge opioids after TKR.  This study has the potential spur further interest and resources toward disparities in pain management and is in line with several national and local public health initiatives.  In addition, this article has the potential to spark interest in further research in this domain.

 The greatest strengths of this study are its originality and its potential impact the health policy, the allocation of health care resources towards eliminating health disparities, and

 future research.

 The greatest weakness of this study is the use of secondary data, that was not collected for the purpose of this study, resulting in the potential for bias.  For example, other variable, such has history of substance abuse, urine drug screens, and other known determinant of opioid prescribing behavior was not included in the analysis.  Not including such variables could result in selection bias and or confounding.  For example, it has been shown that African Americans have been shown to be more often diagnosed with alcohol use disorder when compared to whites even when drinking the same amount of alcohol ^1^.  This could certainly confound the relationship between race and post discharge opioid prescribing.  However, this topic does not significantly limit the conclusions drawn by this study.  In fact, it further describes the presence of health disparities in the VA system and how health disparities result in a domino effect, whereby an act of disparate care can produce another.  Nevertheless, future studies in this domain might include such variables.

Is the work clearly and accurately presented and does it cite the current literature?

Yes

If applicable, is the statistical analysis and its interpretation appropriate?

Yes

Are all the source data underlying the results available to ensure full reproducibility?

Yes

Is the study design appropriate and is the work technically sound?

Yes

Are the conclusions drawn adequately supported by the results?

Yes

Are sufficient details of methods and analysis provided to allow replication by others?

Yes

Reviewer Expertise:

Chronic pain, opioids, acute pain, population health, health disparities

I confirm that I have read this submission and believe that I have an appropriate level of expertise to confirm that it is of an acceptable scientific standard.

## References

[1] : Racial and Ethnic Bias in the Diagnosis of Alcohol Use Disorder in Veterans. *Am J Psychiatry* .2023;180(6) : 10.1176/appi.ajp.21111097 426-436 10.1176/appi.ajp.21111097 37132202 PMC10238581

# Reviewer report: Reviewer response for version 1

**DOI:** 10.5256/f1000research.152981.r306079

**Published:** 2024-08-21

**Authors:** Alethia Sellers

**Affiliations:** 1 Anesthesiology and Perioperative Medicine, The University of Alabama at Birmingham Department of Medicine (Ringgold ID: 164494), Birmingham, Alabama, USA

This retrospective study identified age and racial disparities in the use of regional anesthesia and opioid prescriptions on discharge for Atlanta Veterans Affairs Health Care System patients undergoing total knee arthroplasties. The methods and analysis were detailed enough for this retrospective study to be replicated at another institution. This article is novel in its review of discharge opioid prescribing and use of regional anesthesia. It details how bias may have affected the outcomes found. It provides some tangible suggestions for improvement to reduce the disparities based on current literature via a modified socioecological approach. This also provides some basis of need for a larger retrospective study of the Veterans Health Administration overall.

 Introduction

 -Substantial evidence was presented to support that there is biased pain care in the United States that result in disparity.

 - Although not necessary, it might have been helpful to provide a sentence with the example(s) of the limited research exploring disparities in postoperative pain prescriptions and visualize how this current research study filled in the gap.

 -The hypotheses were clearly stated, appropriate in depth and specificity.

 Methods

 -The inclusion and exclusion criteria were indicated clearly with further explanations of why as it relates to the Atlanta VA.

 -It was helpful to include the CPT codes used to query the medical record and allow for the study to be reproduced if needed at another institution.

 -The table for the opioid conversion factor provided further clarification for the oral morphine equivalents and was well received within the article.

 -The statistical analysis was communicated very clearly including why certain data was used or not used.

 Results

 -The tables provided a very clear visual of the results from the statistical analysis in addition to the summaries.

 Discussion

 -The discussion was very well written beginning with great explanations for the results seen for the regional anesthesia and post-operative discharge opioids. 

 - Particularly powerful in the discussion was the Modified Socioecological Approach to Disparities in Pain Care.

 - For first readers of medical disparities, the breakdown of each level in this approach provides specific and detailed explanation of why there is a disparity and action items to improve or reduce them.

 - As well as for the readers well versed in medical disparities, the details in the explanation of the approach provides great action items to use to continue to reduce disparities.

 - In the individual to patient explanation, the authors may have also considered adding the disparity example of the Tuskegee experiment as well for distrust reasons.

 - The authors were very realistic in their study limitations. However, the study and article overall was very well written.

 -For future directions, it would be ideal to report back the comparison of the same parameters for 1-2 years after the multimodal analgesic regimen for TKA patients was implemented to help determine the impact on the disparities explored in this article.

Is the work clearly and accurately presented and does it cite the current literature?

Yes

If applicable, is the statistical analysis and its interpretation appropriate?

Yes

Are all the source data underlying the results available to ensure full reproducibility?

Yes

Is the study design appropriate and is the work technically sound?

Yes

Are the conclusions drawn adequately supported by the results?

Yes

Are sufficient details of methods and analysis provided to allow replication by others?

Yes

Reviewer Expertise:

Acute Pain Medicine, Chronic Pain Medicine, Interventional Pain, Quality Improvement and Patient Safety, Medical Education, Medical Disparities, Pain Disparities

I confirm that I have read this submission and believe that I have an appropriate level of expertise to confirm that it is of an acceptable scientific standard.
